# Of herds and societies—Seasonal aspects of Vinča culture herding and land use practices revealed using sequential stable isotope analysis of animal teeth

**DOI:** 10.1371/journal.pone.0258230

**Published:** 2021-10-07

**Authors:** Rosalind E. Gillis, Jelena Bulatović, Kristina Penezić, Miloš Spasić, Nenad N. Tasić, Cheryl A. Makarewicz

**Affiliations:** 1 Faculdade de Ciências Humanas e Sociais, ICArEHB, Universidade do Algarve, Faro, Portugal; 2 Graduate School “Human Development in Landscapes”, Kiel University, Kiel, Germany; 3 Faculty of Philosophy, Laboratory for Bioarchaeology, University of Belgrade, Belgrade, Serbia; 4 BioSense Institute, University of Novi Sad, Novi Sad, Serbia; 5 Department of Archaeology, Prehistoric Collection, Belgrade City Museum, Belgrade, Serbia; 6 Faculty of Philosophy, Department of Archaeology, University of Belgrade, Belgrade, Serbia; 7 Institute of Prehistoric and Protohistoric Archaeology, Kiel University, Kiel, Germany; University at Buffalo - The State University of New York, UNITED STATES

## Abstract

Late Neolithic Vinča communities, spread over much of central and northern Balkans during the late sixth to mid-fifth millennium BC and characterised by unusually large and densely population centres, would have required highly organised food production systems. Zooarchaeological analysis indicates that domesticate livestock were herded, but little is known about the seasonal husbandry practices that helped ensure a steady supply of animal products to Vinča farming communities. Here, we present new stable carbon (δ^13^C) and oxygen (δ^18^O) isotopic measurements of incremental bioapatite samples from the teeth of domesticated livestock and wild herbivore teeth from two late Neolithic Vinča culture sites: Vinča-Belo brdo and Stubline (Serbia). Our results show a low variation overall within sheep and goats in terms of pasture type that may have been composed of seasonal halophyte plant communities, which have higher δ^13^C values due to the saline rich growing environments. Cattle feeding strategies were more variable and provided with supplementary forage, such as cut branches or leafy hay, during winter. The sharp distinction in the management of cattle and sheep/goat may be associated with the development of herding strategies that sought to balance livestock feeding behaviours with available forage or, more provocatively, the emergence of household-based control over cattle–an animal that held a central economic and symbolic role in Vinča societies.

## Introduction

During the Late Neolithic (5300/5200 to 4600/4500 cal BC), much of the central Balkans and southeast parts of the Vojvodina and Transylvania regions were occupied by Vinča communities which produced dark burnished ceramics and figurines often bearing unique triangular faces [1,2]. Compared with earlier Starčevo-Körös-Çriş cultural complex, Vinča groups occupied a diverse range of site types, including tells, with long occupation sequences [3]. Excavations of settlements have revealed extensive organisation of social space, with designated central places and well-defined lay-out of houses including regular spaced thoroughfares [4–7]. Population estimates based on the number of ‘house’ structures in Vinča culture settlements suggests these sites were densely populated with up to several hundred inhabitants (c.*f*. Porčić [8]). The increase in settlements distribution and size suggests a population increase that was supported by herding and agricultural activities. Vinča settlements were often situated on the alluvial plain and lower terraces of the Danube, Sava, Morava, and their tributaries, and subsistence systems shaped by periodic flooding of the landscape which would have provided rich seasonally replenished alluvial deposits ideal for cropping production [9]. Crop growing may have been limited to small plots that were intensively cultivated as suggested by unpublished analysis of weed seed assemblages (Filipović pers. comm). Heavier soils of the wooded hill slopes were probably unlikely to have been cultivated.

Vinča communities exploited a wide spectrum of domesticated plants including einkorn (*Triticum monococcum*), emmer (*Triticum dicoccum*) and barley (*Hordeum vulgare*, hulled and naked), as well as pulses such as lentils (*Lens culinaris*) and pea (*Pisum sativum*) [10]. Domesticated cattle (*Bos taurus*) and pigs (*Sus scrofa*) increased in socio-economic importance over time within Vinča communities, likely due to intensification in meat and dairy production associated with a diachronic increase in settlement size and populations [11]. The high proportion of adult cattle identified at the late Vinča sites, Divostin and Gomolava, for example, may indicate dairying and use of animal traction [12,13], while at other sites, cattle appear to have mainly exploited for their meat indicated by the selective slaughter of sub-adult animals (18–36 months) [11,14–19]. Provisioning Vinča communities with an adequate supply of animal products would have required a high level of animal maintenance to ensure herd growth and productivity, in particular a sufficient source of good pasture and forage. Vinča herders may have employed at various times within the seasonal cycle complementary strategies that served to further provision their hungry herds by leading animals to crop stubble, or provisioning them with cut branches or leafy hay [20] collected from woodlands as has been previously hypothesised for Vinča domesticated pigs and cattle [21,22]. Waterlogged pastures caused by seasonal river flooding may have encouraged the development of mobile herding strategies where animals were moved to different, drier pastures within the seasonal calendar [11,21].

Carbon and nitrogen stable isotopes can be used to investigate husbandry practices, including forage and pasture management strategies [23–27]. The δ^13^C signatures of animal tissues reflect the carbon component of protein (bone) and carbohydrate (fats) intake, and total diet (teeth) and, for herbivores, tissue carbon isotope ratios directly reflect plant stable isotopic composition [28]. Thus can be used to reconstruct pasture and fodder resource environments [23–25]. The nitrogen isotopic composition of bone and dentine collagen can indicate the level of anthropogenic activity associated with their food source [22,23,26,27]. Previous stable isotopic analyses of bone collagen from faunal material recovered from Stubline and Vinča-Belo brdo suggest that different types of forage were provisioned to different domesticated species, with cattle exhibiting a wide range of δ^13^C values indicative of open and closed forest pasturing [22]. However, stable isotope values measured from bone collagen represent an integrated signal representing a long-term ‘dietary average’ and, due to constant bone remodelling, do not capture seasonal-scale shifts in dietary intake. Sequential sampling of tooth enamel captures at high-resolutions carbon and oxygen isotopic change especially in species bearing hypsodont teeth [29,30]. At mid- and high- latitudes, the stable oxygen isotope ratios of precipitation are strongly correlated to mean annual ambient temperature with low δ^18^O values expressed during the winter months and higher values during the summer [29,31]. Consequently, oxygen isotopes measured from incrementally sampled teeth provide a seasonal-scale temporal framework that assists in the interpretation of δ^13^C values that may be related to seasonal variation in pasture and fodder sources.

We present isotopic results measured from the bioapatite fraction of incrementally sampled teeth from domesticated and wild herbivores to further explore the Late Vinča fodder and pasture management strategies. These results are further contextualized within published stable isotopic results from bone collagen samples [22].

## Cultural and climatic context

The epicentre of the Vinča culture was located in Serbia and dispersed throughout the river valleys of the northern and central Balkans, including southern–most Hungary and parts of the Transylvania. Vinča culture communities also occupied hilltop settlements including, for example, Gradac, Valač, Pljosna Stijena, Gradina-Stapari [32], while numerous settlement located in wetland and marshland landscapes have been identified in western Serbia. Cave sites are also known, including the sites of Petnica in western Serbia and Nandru Vale in Romania [33]:45]. As part of this study and Gillis, Bulatović [22], we have focused on faunal material recovered from assemblages that date to the terminal phase of Vinča culture, *i*.*e*. Vinčа D/Vinča–Pločnik II phase (between 4850 and 4650/4600 cal BC [2,34]). On the broader regional scale, this phase is characterised by several major shifts in settlement organisation, material culture production, such as new vessel forms [35–39] and standardisation of ceramic production processes [40], and specialised animal economies. The distribution of similar material culture objects across the Vinča macro-region indicates the establishment of large-scale social networks within existing inter-regional trade/exchange routes [1,41]. The late Vinča phase also witnessed the widespread manufacture of copper items within communities located in the eastern and southern parts of Serbia [42–44], as well as the long-distance transmission of finished copper objects which were distributed over the almost entire area of the Vinča cultural sphere [2,45]. Overall, late Vinča settlements appear to be much larger in size and more complex in organisation than the earlier ones, consisting of up to several hundred elaborately built and equipped houses [5,46,47], complex of ditches surrounding the settlement [1,5], and supporting several thousand inhabitants [48].

**Fig 1:**
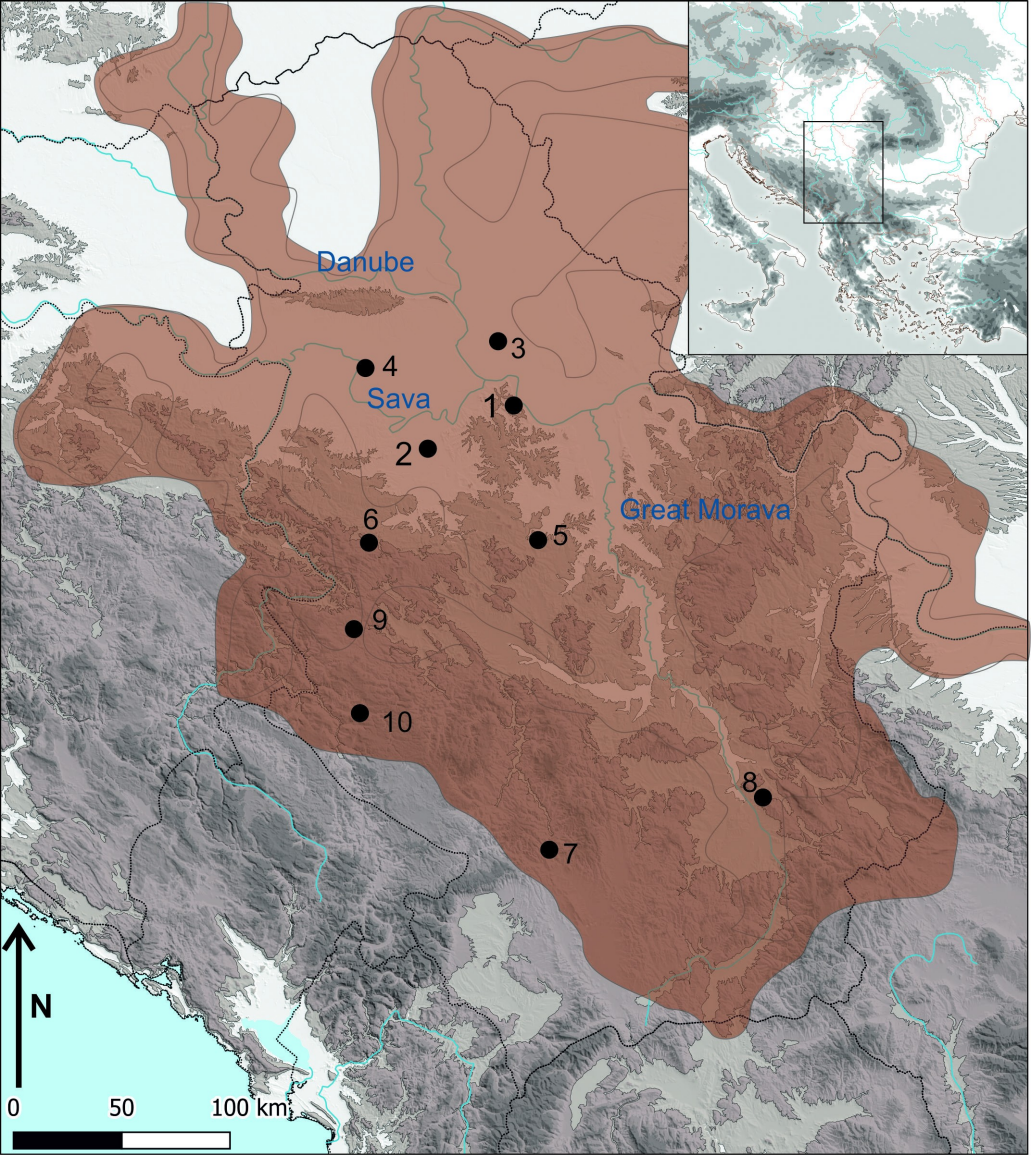
Map of the study sites with Vinča distribution and sites mentioned in the text. 1. Vinča-Belo brdo; 2. Stubline; 3. Opovo; 4. Gomolava; 5. Divostin; 6. Petnica; 7. Valač; 8. Gradac; 9. Gradina-Stapari; 10. Pljosna Stijena. The base map is available under the CC4.0 at the *U*.*S*. *Geological Survey*. (Credit: Penezić/Pendić).

We analysed material from two settlements, Vinča-Belo brdo and Stubline, both located on the alluvial plains and lower terraces of the Danube and Sava respectively, and their tributaries in northern Serbia (Fig 1). The prominent tell site of Vinča-Belo brdo stands several meters high on the southern bank of the Danube. The site has suffered erosion due to the Danube and covers an area of approx. 12 ha [49]. The Neolithic Vinča-Belo Brdo sequence represents a long occupational history [50], established by Starčevo groups (*c*. 5700–5300 cal BC) and followed by Vinča culture groups until *c*. 4550 cal BC when occupation ceased [51,52]. Recent excavations have focused on the final Vinča occupation, represented by two successive horizons of burnt rectangular buildings, interspaced with a horizon of unburnt buildings, oriented along a north-west axis, composed of two to three rooms, and housing at least one kiln per structure, with some buildings containing several kilns [52]. During the Vinča D period, the external space between houses was up to 2 m in places. Several communication lines (“streets”) were placed between houses in some areas, with no open spaces within settlements have been identified, suggesting that animal pens, gardens or cultivation areas were external to settlements during the late Vinča phases. Next to Vinča-Belo brdo is the Bolečica river, a tributary of the Danube, which annually floods part of the landscape surrounding the site [21]. Damp alluvial soils in the river floodplain would have supported gallery forests and wetland vegetation, such as willow *sp*. (*Salix sp*.) and reeds (*Phragmites communis*). Thermophilous taxa, particularly oak, grew on rich chernozem soils and dominated forests that spread over hilltops and slopes [21].

The site of Crkvine at Stubline (herein Stubline) is located *circa* 50 km east of Vinča–Belo Brdo (Fig 1). In a broader geographical context, Stubline lies in the micro-region called Drenski Vis, located in the Posavina-Tamnava region. Approximately 12.5 ha in size, Stubline is situated on an elongated plateau of about 23.6 ha (100 m a.s.l.) [5,6,46,53,54]. The plateau was surrounded by two watercourses to the north and south, that meet at the break of slope in the south-western part of the settlement. Based on the geophysical survey, the earliest settlement consisted of about 120 houses surrounded by a system of double ditches irregular in plan. Over time, the Vinča community grew to occupy almost the entire eastern portion of the plateau and may have consisted of more than two hundred houses [54]. At least two occupation phases have been so far identified at Stubline: Stubline Ia (Vinča D1 phase in the eastern part of the plateau) and Stubline Ib (Vinča D2 phase throughout the whole plateau) [54]. A relative chronology based on material culture indicates that the Vinča culture occupation spanned a period of around 200 to 250 years (4850/4800–4650/4600 BC) [54].

The climate today is characterised as humid sub-tropical (Cfa) according to the Köppen climate classification [55]. The warm season lasts on average for 3–4 months beginning in May with the average temperature reaching 24°C while the cold season last for on average for 3 months with temperatures falling to -2°C. Precipitation falls mainly in late spring and late autumn (c. 70mm), with most of the precipitation falling as snow (20-8mm) during the cold season [56]. The climate during the occupation of the sites was wetter and warmer in the winter than today, with reduced variation between seasons [57].

## Material and methods

### Sample selection

All animal bone and teeth from Vinča-Belo brdo and Stubline were sampled by REG, assisted by JB and KP, with the permission of the local authorities and excavators (MS, NT). The collections are housed in City Museum, Belgrade. Details of the sampling protocol for wild and domesticated faunal bones for stable isotopic analysis of bone collagen are outlined in Gillis, Bulatović [22]. The complete sample list is provided in S1 Table. The Vinča-Belo brdo herbivore tooth specimens were recovered from archaeological features (e.g., pit-holes, demolition layers) and cultural layers dated to the latest occupation phase (Vinča D2 cultural phase; 4700–4550/4500 cal BC [52]. Specimens from Stubline were from archaeological features (e.g. waste pits, ditches) and cultural layers dated to the Vinča D period.

To examine the seasonality of husbandry practices, we selected teeth from cattle, sheep/goat, and domesticated pig, based on state of occlusal wear and overall specimen preservation. The third mandibular molars of domestic cattle (*Bos taurus*; Vinča-Belo brdo n = 5, Stubline n = 1) and sheep/goats (*Ovis aries/Capra hircus*; Vinča-Belo brdo n = 4, Stubline n = 5) were selected for analysis. The third molar in both species begins development around 10 months and erupts around 24 months [58,59]. We focused on teeth with dental wear up to stage G for both sheep/goats and cattle [60], in order to increase the chances of capturing a full seasonal cycle in the isotopic record. The isotopic composition of the third molar reflects adult dietary intake only and is free of ontogenic effects, including suckling and weaning, that influence the carbon and oxygen isotopes of tooth bioapatite.

For domesticated pigs (*Sus scrofa*), 3 individuals from both sites were sampled with both the mandibular M2 and M3 selected. The bunodont teeth of pigs display short crowns in comparison to ruminants. Enamel mineralisation of the lower M2 in modern pigs is initiated at around 3 months and finishes around 11 months, while mineralization of the mandibular M3 begins at approximately 11 months and extends through ca. 18 months [61,62]. Pigs are usually weaned by the second month of life [63], and therefore we do not expect the carbon and oxygen isotopic composition of tooth bioapatite to be affected by a suckling signal.

To provide a reference for a ruminant grazing/browsing within a forest environment we sampled two red deer (*Cervus elaphus*) individuals (7237, 7238) from Vinča-Belo brdo, with two teeth from a single individual (7237-M2/M3). Red deer mandibular M2 mineralisation begins around 4 months, around when weaning occurs, with the crown complete at 9 months. For the M3, mineralisation begins around 13 months with completion of the crown at 26 months based on a study on modern red deer from Richmond Park, London [64].

### Stable isotope sampling and analysis methodology

Prior to sequential enamel sampling, the tooth surface was cleaned using an abrasive drill bit. A sequence of enamel samples was then drilled on the buccal surface from the enamel-root junction (ERJ) to the apex of the crown. Purification protocol for stable isotopic analysis followed [65]. Purified bioapatite samples of domesticated samples were weighed between 550–650μg were analysed on a Kiel IV device interfaced to a Delta V Advantage IRMS at SSMIM platform, Paris. The analytical precision estimated from average of the internal standards (Marbre LM normalized to NBS 19: between four and eight per run) for δ^13^C analyses was 0.02‰ (range: 0.009–0.05‰) and for those of δ^18^O was 0.04‰ (range: 0.03–0.06‰). The red deer samples were analysed at the Leibniz labor at Kiel University (Germany). The analytical precision was for δ^13^C analyses was 0.02 (range: 0.01 to 0.03) and δ^18^O was 0.03‰ (range: 0.01 to 0.04).

### Stable isotopic enrichment between diet and bioapatite

Studies concerning the enrichment of δ^13^C values between diet and bioapatite have shown that it can vary between species depending on dietary composition, physiology, and body size [30,66–69]. The magnitude of the enrichment depends on the plant composition (C_3_ or C_4_ plants) of an animals’ diet [67–69], and intra-tooth variation in the enrichment factor has been observed where the diet composition changes during the tooth’s development [69]. We have chosen to use a single enrichment value has been used for all species of 14.1‰ [66] as applied previously in Balasse, Bălăşescu [70] for the analysis of Romanian Late Neolithic and Chalcolithic settlements.

### Correction of modern stable isotopic data

The burning of fossil fuels and to a lesser extent, the removal of large stretches of forests, has contributed to changes in the carbon isotope composition of atmospheric CO_2_. We applied a correction factor of 1.7‰ to carbon isotope data obtained from modern tissues. This calculation is based on the δ^13^C value of CO_2_ at present (−8‰) and the given studied period. The δ^13^C value of CO_2_ during the Late Vinča period was −6.3‰ based on interpolated data from Antarctic ice-cores [71].

### Statistical analysis

The summary statistics used were the average midpoint value, range, and amplitude per species. The midpoint was calculated *(δ*^*xx*^*X*_*Max*_*+ δ*^*xx*^*X*_*Min*_*)*/2, and then an average and standard deviation was calculated per species. The amplitude was calculated by subtracting the *δ*^*xx*^*X*_*Min*_ from the *δ*^*xx*^*X*_*Max*_, an average was then calculated per species. The maximum and minimum value per species is true range and not an average.

## Results

### Stable oxygen isotope results

The δ^18^O values of Vinča-Belo brdo cattle average mid-point was −7.2‰ (range: −9.6 to −4.2‰) (Table 1). In comparison, the single Stubline cattle averaged −8.5‰ (range: −10.3 to −6.8‰). Vinča-Belo brdo sheep/goat specimens exhibited an average of −6.1‰ (range: −10.5 to −1.1‰) like those from Stubline (average −6.5‰, range: −10.6‰ to −2.4‰). The δ^18^O values exhibited by domesticated pigs from both sites fall within the range of −9.1‰ to −6.3‰, with the greater variation evident in Vinča-Belo brdo pigs, particularly the M2s (Table 1). Red deer δ^18^O values ranged from −9.0‰ to −4.6‰, which is comparable to variation exhibited in cattle teeth.

**Table 1:** Summary of δ^18^O values from the incremental analysis of cattle (*Bos taurus*), sheep/goat (*Ovis aries/Capra hircus*), pig (*Sus scrofa*), and red deer (*Cervus elaphus*). Results for individual teeth are in S2 Table.

Species	n	Average δ^18^O_midpt_ (‰)	Average Amplitude	δ^18^O_min_ (‰)	δ^18^O_max_ (‰)
Vinča-Belo brdo					
*Bos*	5	−7.2	3.4	−10.3	−4.7
*Ovis/Capra*	4	−6.1	5.6	−10.5	−1.1
*Sus* M2	3	−7.6	0.6	−9.1	−6.3
*Sus* M3	3	−7.4	0.8	−7.9	−7.3
*Cervus* M2	1	−6.8	3.8	−8.7	−4.9
*Cervus* M3	2	−6.5	2.7	−9.0	−4.6
**Stubline**					
*Bos*	1	−8.5	3.5	−10.3	−6.8
*Ovis/Capra*	5	−6.5	6.1	−10.6	−2.4
*Sus* M2	3	−7.5	0.7	−9.1	−6.9
*Sus* M3	3	−7.4	0.5	−8.0	−6.9

### Stable carbon isotope results

The δ^13^C values from bioapatite samples of domesticated animals ranged from −14.1‰ and −7.9‰ with the samples from Vinča-Belo brdo cattle exhibiting the greatest range in values observed from all species (Table 2). Cattle from Vinča-Belo brdo had an average mid-point carbon isotope value enriched +1.5‰ in 13C relative to other domesticated species. Values from a single cattle tooth from Stubline fell within the range exhibited in cattle from Vinča-Belo brdo. The δ^13^C values for Vinča-Belo brdo sheep/goat teeth range between −14.1‰ and −10.1‰, with the average midpoint value of −12.0‰, while at Stubline, sheep/goat teeth exhibited a slightly larger range in values (−14.5‰ to −8.9‰). The M2s from domestic pigs at Vinča-Belo brdo exhibit values that were on average 0.3‰ higher midpoints values than M3s. At Stubline, there was little difference between the average midpoints from M2s and M3s of domesticated pigs (M2: −12.5‰; M3: −12.6‰).

**Table 2:** Summary of δ^13^C results from the incremental analysis of cattle (*Bos taurus*), sheep/goat (*Ovis aries/Capra hircus*), pig (*Sus scrofa*), and red deer (*Cervus elaphus*) mandibular teeth. Results for individual teeth are presented in S3 Table.

Species	n	Ave. δ^13^C_mdpt_ (‰)	Average amplitude	δ^13^C_min_ (‰)	δ^13^C_max_ (‰)
Vinča-Belo brdo					
*Bos*	5	−10.8	1.6	−14.1	−8.0
*Ovis/Capra*	4	−12.0	2.5	−14.1	−10.1
*Sus* M2	3	−12.1	0.7	−13.1	−11.5
*Sus* M3	3	−11.8	1.3	−12.2	−12.0
*Cervus* M2	1	−13.3	4.6	−15.6	−10.9
*Cervus* M3	2	−12.7	1.6	−13.7	−11.9
Stubline					
*Bos*	1	−11.0	1.3	−11.6	−10.4
*Ovis/Capra*	5	−12.2	3.4	−14.5	−8.9
*Sus* M2	3	−12.5	0.3	−13.0	−12.2
*Sus* M3	3	−12.6	0.4	−12.8	−12.0

The two red deer individuals exhibited a large range in values (−15.6‰ to −10.9‰; Table 2). The second molar from red deer 7237 yielded δ^13^C values ranging from −15.6‰ to −10.9‰, with the lowest midpoint of the entire dataset of −13.3‰ while the M3s from the same individual and 7238, exhibited a smaller range in values (−13.7‰ to −11.9‰) and an average midpoint of −12.7‰.

**Fig 2:**
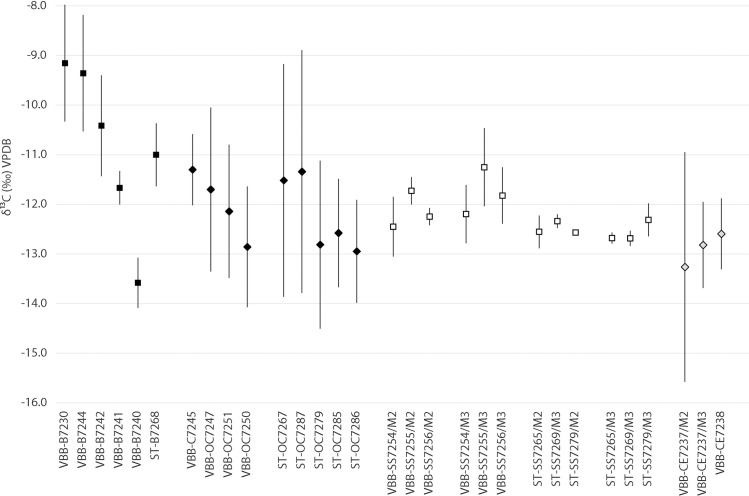
Comparison of δ^13^C_ave_, δ^13^C_max_ and δ^13^C_min_ from incremental samples. Cattle (B), domestic pigs (SS), sheep/goat (OC) and red deer (CE) teeth from Vinča-Belo brdo (VBB) and Stubline (ST).

Amplitude of intra-tooth carbon isotope change provides insight into isotopic variation in forage ingested by animals. The average amplitude for cattle from Vinča-Belo brdo was 1.7‰ (range: 0.7 to 2.4‰) with some individuals (*Bos* 7240 and 7241) exhibiting an amplitude of intra-tooth isotopic change of less than 1‰ to 2.4‰ (*Bos* 7230, Fig 2). At Vinča-Belo brdo, sheep/goat teeth exhibit the largest amplitude in intra-tooth isotopic change, averaging 2.5‰ in Δ^13^C (range: 1.4 to 3.3‰). In comparison, the single goat identified in the Vinča-Belo brdo assemblage (O/C 7245) exhibited a low amplitude of intra-tooth change (1.4‰). Two sheep/goat individuals (7267 and 7287) had the largest intro-tooth amplitude of 4.7‰ and 4.9‰ respectively. Overall, sheep/goat samples from Stubline exhibit the largest average amplitude of change for domesticated animals in the dataset (3.4‰). Pig intra-tooth amplitude is greatest at Vinča-Belo Brdo (0.7–1.3‰) compared to those from Stubline (0.3–0.4‰). Red deer M3s exhibit an average amplitude of 1.6‰, while the single M2 has the largest amplitude in the dataset of 4.6‰.

### Combined incremental results

**Fig 3:**
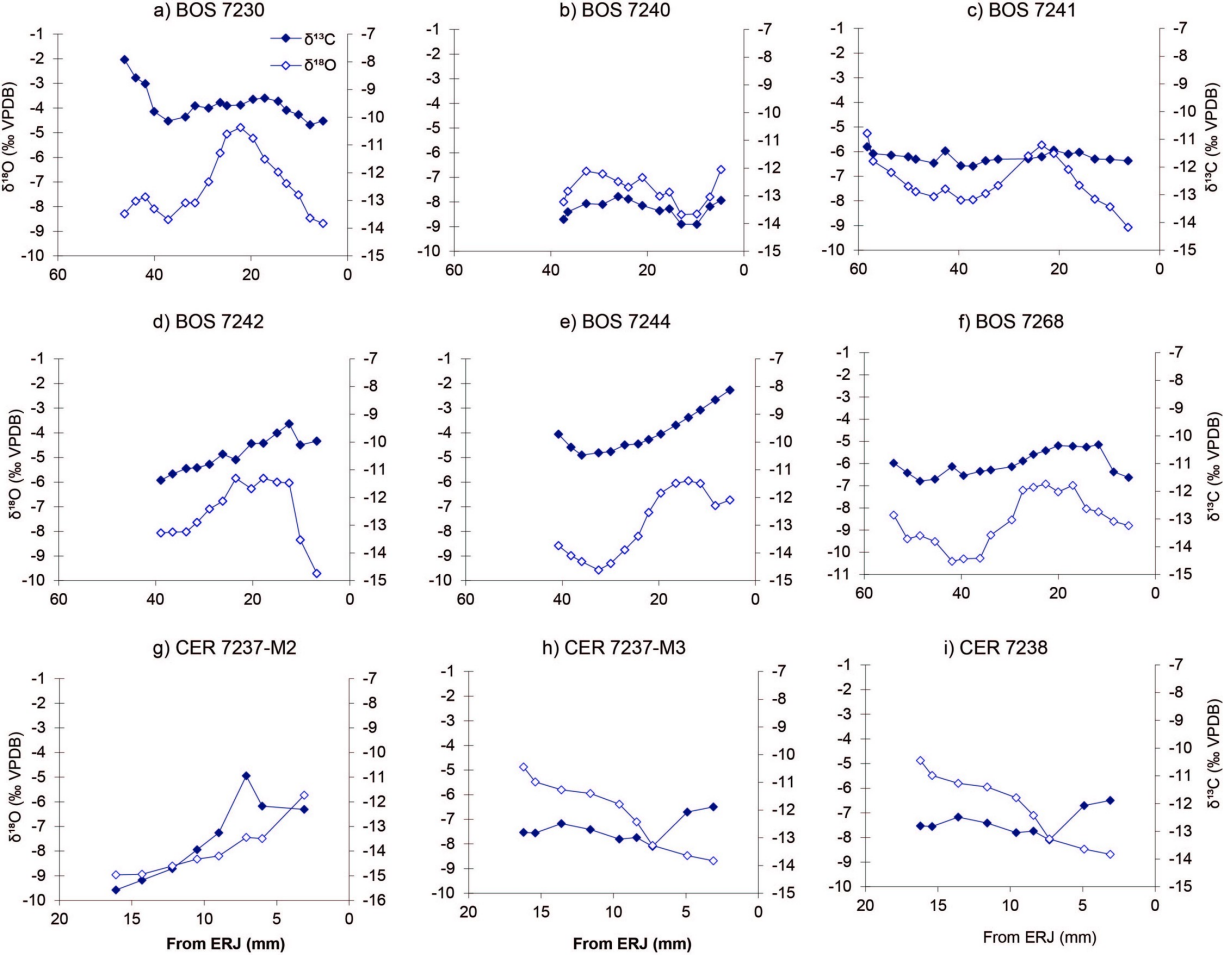
δ^13^C (filled diamond) and δ^18^O (open diamond) values for individual cattle teeth. Insets: a) Bos 7230, b) Bos 7240, c) Bos 7241, d) Bos 7242, e) Bos 7244 (Vinča-Belo brdo), f) Bos 7268 (Stubline), g) Cervus 7237-M2, h) Cervus 7237-M3 and i) Cervus 7238-M3. ERJ is the enamel root junction.

For cattle, there is considerable variation in the relationship between δ^18^O and δ^13^C values. Most individuals follow a pattern where δ^13^C values increase during the summer months (Fig 3B and 3D–3F). BOS 7241 and the single tooth from Stubline (BOS 7268) exhibits little variation over the seasonal cycle (Fig 3C and 3F). While BOS 7244 and 7230, the δ^13^C values remain high during winter when δ^18^O values decrease. The red deer teeth also show a similar decrease in δ^13^C values during the winter; this is particularly evident in 7237-M2. In contrast, carbon isotopic values increase in 7237-M3 when δ^18^O values decrease, i.e., during winter (Fig 3G–3I). For domesticated pigs analysed, little variation in δ^13^C values was observed reflecting in part the bunodont tooth geometry.

**Fig 4:**
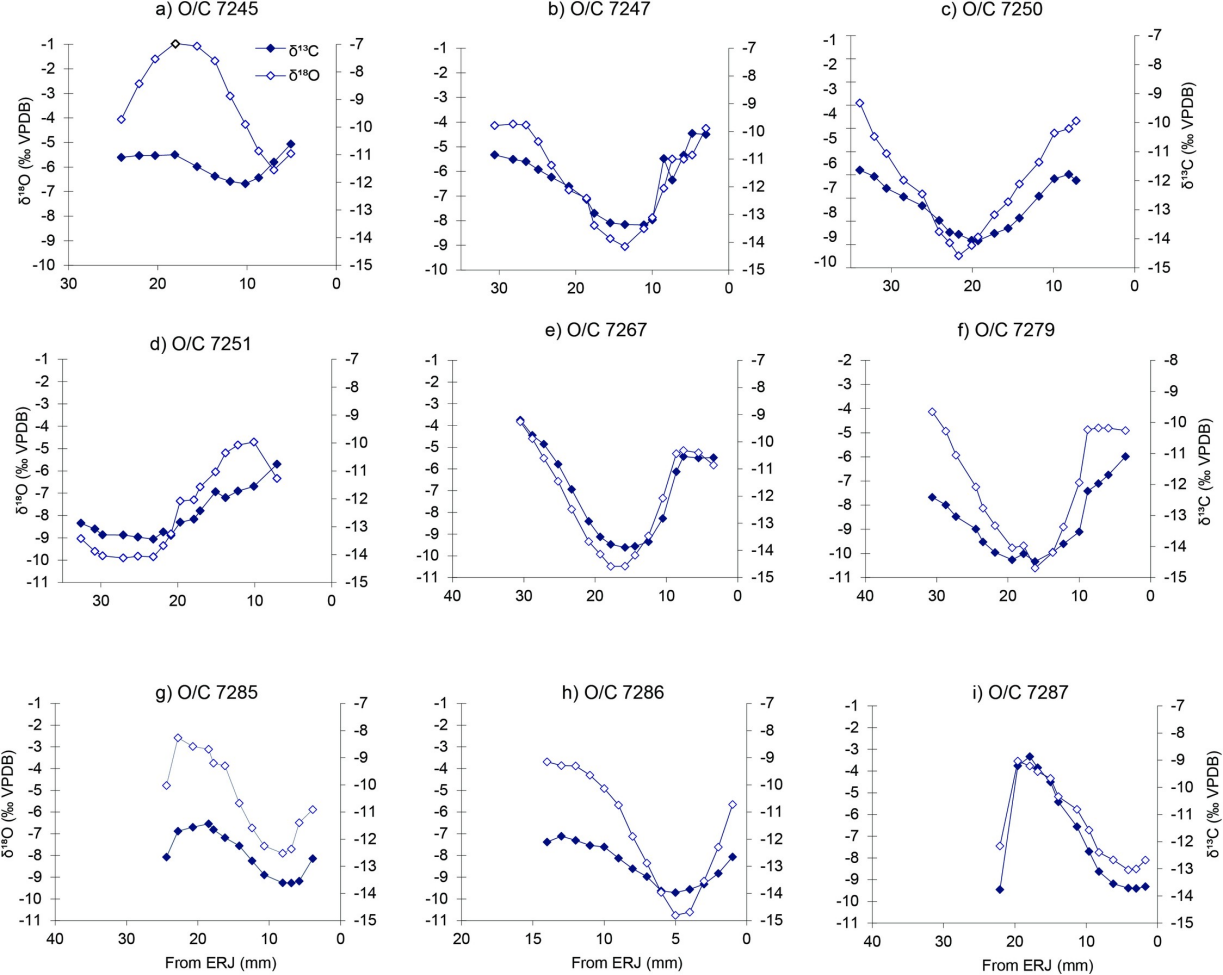
δ^13^C (filled diamond) and δ^18^O (open diamond) values for individual sheep/goat M3s, Vinča-Belo brdo. Insets: a) O/C 7245, b) O/C 7247, c) O/C 7250, d) O/C 7251, and Stubline e) O/C 7267, f) O/C 7279, g) O/C 7285, h) O/C 7286 and i) O/C 7287. ERJ is the enamel root junction.

Within our study, sheep/goat individuals from both sites exhibited a strong correspondence between δ^18^O and δ^13^C values, high δ^13^C values during summer and vice a versa (Fig 4) except for the single goat (O/C 7245, Fig 4A). In this individual, low δ^13^C values (> −11.9‰) coincide with high summer season δ^18^O values.

## Discussion

Previous stable carbon and nitrogen isotopic analysis of bone collagen samples from the Late Vinča phases (4800 to 4600 BC) at Vinča-Belo brdo and Stubline pasture and foddering practices for sheep, goat and cattle [22]. The large range of δ^13^C values from bone collagen from domestic cattle at Vinča-Belo brdo suggests they may have been grazed across different environments (water meadows, open grassland, open/closed forest). While the relative low bone collagen δ^13^C values at Stubline may indicate that they were kept mainly within wooded or waterlogged environments. At both sites, caprines and domestic pigs consumed a diet enriched in ^15^N, which strongly suggests these animals were raised on either crop residue from cultivars raised on manured soils or pastures repeatedly visited by livestock [23]. We previously proposed this supports the hypothesis that caprines herds and pigs were fed crop by-products while penned within the settlement [11], with access to the seasonal flooded landscape limited by herders to reduce foot rot in livestock [21]. The δ^13^C and δ^18^O values from incremental bioapatite samples presented here provides us with the opportunity to test these hypotheses through integration of seasonal short-term stable isotopic records with those from bone collagen.

### Oxygen isotopes of obligate and non-obligate drinkers

**Fig 5:**
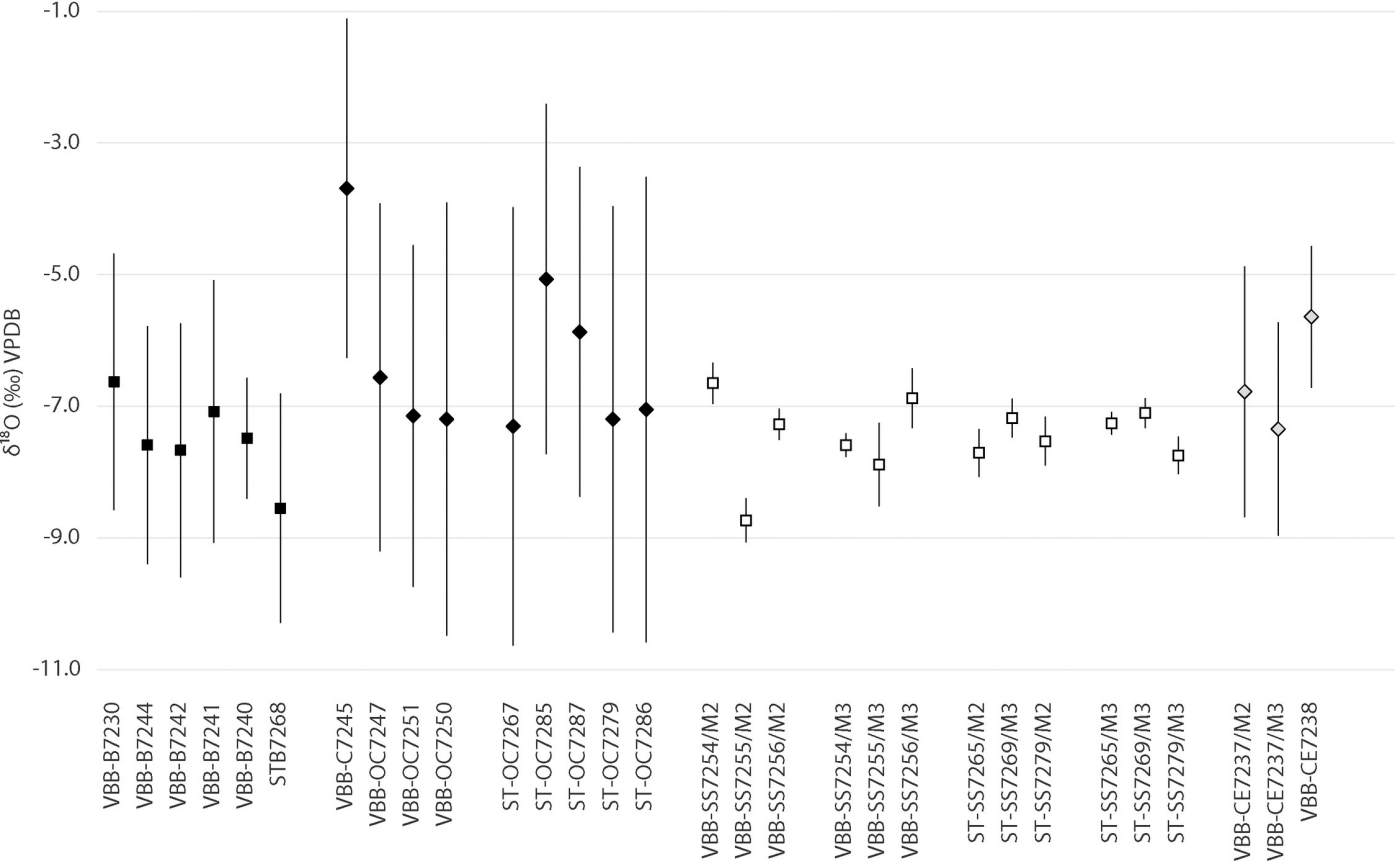
Comparison of δ^18^O_ave_, δ^18^O_max_ and δ^18^O_min_ for incremental samples. Cattle (B), pigs (SS), sheep/goat (OC) and red deer (CE) teeth from Vinča-Belo brdo (VBB) and Stubline (ST).

There were clear differences between ruminants and omnivores in their oxygen isotope observed at both sites. Sheep/goat samples from Vinča-Belo brdo and Stubline exhibit the largest average intra-tooth amplitude observed in domestic livestock, averaging 5.6‰ and 6.1‰, respectively for Vinča-Belo brdo and Stubline. The average amplitude per tooth for cattle from both sites were similar (Vinča-Belo brdo: 3.4; Stubline: 3.5‰), and close to that observed in deer (M2: 3.8‰; M3: 2.7‰), with domestic pigs exhibiting the lowest amplitude (<1 ‰; Fig 5). The oxygen stable isotopic composition of bioapatite is in near isotopic equilibrium with the oxygen isotopes of body water [72]. These isotopic ratios are influenced by those of imbibed water, which is in turn affected by meteoric water δ^18^O values, which is influenced by altitude, local humidity, and temperature [29,73]. Drinking water source type, also influences body water oxygen isotope ratios. Groundwater typically represents a local meteoric water δ^18^O average, while rivers will vary seasonally depending on the input of melt water. Leaf water is enriched in ^18^O in comparison to the local average due to evapotranspiration [74]. Here we discuss the differences in the midpoint value and intra-tooth amplitude between the maximum and minimum δ^18^O values between species. These will provide us an insight into water consumption habits of domesticated animals.

The large amplitude of intra-tooth oxygen isotopic change observed in sheep/goat may be due to ingestion of 18O enriched leaf water which comprises a large proportion of total body water in caprines [75]. Leaf water oxygen isotope ratios are influenced by the local precipitation d18O values, aridity level, and evaporation rates so that leaf water is enriched in ^18^O relative to precipitation [76]. Consequently, the δ^18^O record of semi-obligate species, which include caprines, is more sensitive to aridity [75]. Serbia is characterised by a marked continental semi-arid climate, with high summer and low winter temperatures. Sheep and goat ingesting leaf water in arid and semi-arid environments may exhibit a large intra-tooth amplitude between high and low values within a single tooth due to animals obtaining water during the summer from leaf water [77]. Our results chime with those previously seen in sheep/goat (4.8‰) from Popină Borduşani (5^th^ millennium BC, Romania) located on the Danube [78].

Cattle, deer, and pigs are obligate drinkers, and it has been shown that leaf-water has limited effect on the δ^18^O values expressed in these species [75]. The composition and seasonal variation in δ^18^O values of water sources is dependent on the frequency of a body of water is fed by sources, such as melt water or tributaries [79]. The seasonal variation within δ^18^O values of standing water, such as lakes and wells, is dampened in comparison to local δ^18^O precipitation because of the effects of evaporation and influx water cancel each other out [80]. In Laurentian great lakes, the seasonal shift in δ^18^O values was less than 2‰ [81]. The amplitude between high and low δ^18^O values within single cattle and deer teeth was greater than 3‰, which is greater than observed by Jasechko, Gibson [80]. It may suggest that cattle and deer ingested water from sources with a greater seasonal variation than observed in Laurentian lakes, like the Danube and the Sava. For pigs, the narrow range may reflect the tooth structure or formation or may reflect pigs ingesting water from standing water sources, such as wells.

The geometry of teeth between species will influence the stable isotopic record. Pig molars have different internal and external dental geometry in comparison to cattle and deer. The mineralisation process is faster in pigs and as with most species the timing of the process is not uniform across a crown [62], which may lead to a dampening of seasonal stable isotopic signal. This may explain the narrow range in oxygen values and low amplitude observed in pigs from this study. Frémondeau, Cucchi [63] demonstrated that incisors are a more suitable tooth for the purposes of capturing seasonality in δ^18^O values, perhaps due to a thinner enamel layers than is seen in molars. Future studies should incorporate incisors to directly investigate pig watering habits. This could potentially open-up new research avenue into analysing differences between species in terms of δ^18^O values to understand how communities may have managed water sources for livestock.

### Sheep/Goat seasonal herding practices

Predicted dietary δ^13^C values from sheep/goat enamel bioapatite samples ranged from −28.6‰ to −23‰, considerably greater than the range previously observed in collagen samples (−26.4‰ to −23.4‰) [22]. A strong synchroneity between the curves of δ^13^C and δ^18^O values, which indicates that that caprines consumed fodder sources that varied considerably in ^13^C over the seasonal cycle. Environmental factors, such as light levels and soil/atmospheric humidity, impact photosynthesis efficiency and respiration in C_3_ plants impact the isotopic fractionation of ^12^C/^13^C [82–84]. We propose three main hypothesis to explain the high variability in livestock δ^13^C values, 1) grazing in marsh land environments; 2) winter grazing in dense non-deciduous forests; 3) supplementary foddering during winter with leafy hay collected from either closed canopy forests during summer when the ‘canopy effect’ would be greatest, or collected from waterlogged galley forests, which also may exhibit low δ^13^C values [85].

The seasonally flooded landscape surrounding the sites may explain the large amplitude of intra-tooth change in δ^13^C values (Vinča-Belo brdo: 2.5; Stubline: 3.4)., which is greater than the values observed in hair samples from modern herbivores grazing on open pastures throughout the year (~1.5‰) [86]. Plants growing in areas subject to seasonal flooding exhibit a strong seasonal variation in foliar δ^13^C values [85] with low carbon isotopes expressed in leaf tissue during flooding. This is caused by a decrease in CO_2_ intake by plants coupled with increased photosynthesis activity results in ^12^C being discriminated over ^13^C [83]. In general, plant stomata during dry periods, particular in regions with hot summers, will close to minimizing water loss and reduce the discrimination against ^13^C, resulting in higher carbon isotopes values in plant tissues. For example, in semi-arid regions where the mean annual precipitation is less than 500mm, the mean value of C_3_ plants, adjusted for the Suess effect, were between −24.7‰ to −23.9‰ [87]. We propose that animals pastured all-year round on a landscape with seasonal flooding under a strong continental climate will exhibit carbon values that reflect a strong seasonal pattern.

The Danube and Sava are active meandering rivers, with paleo-channels that are visible from satellite images. Focusing on Vinča-Belo Brdo, at present the Danube runs close to the site, however in the past it may have flowed further east. There is no evidence of flooding during the occupation levels, which suggests that the site was not as close to the river as today [21]. Areas of standing water or vernal pools may form during flooding within extinct paleochannels [88]. Plant communities in these pools vary in composition over a growing season due to changes in the groundwater levels. Coupled with changes in temperature from spring to summer, these environments may exhibit a large variation in foliar δ^13^C values. These environments contribute to osmotic stress in plants, thus reducing stomatal activity leading to an increase in the uptake of ^13^C increasing overall the foliar δ^13^C values [82]. Therefore, animals grazing on these communities would exhibit δ^13^C values with strong seasonal variation with relatively high values in the summer.

Stubline and Vinča-Belo brdo lies on the edge of the Vojvodina region, which is part of the Pannonian Basin known for its salt steppe, pans, marshes, and lakes [89,90]. Most of these environments are of natural origin and support halophytes plants, such as succulents, xerophytic grasses, and other monocytes, that can tolerate high salt concentrations [89,90]. Drainage and intensive agricultural practices since the 18^th^ and 19^th^ century have led to loss of these environments [91]. Halophyte plant communities exhibit high δ^13^C values due to the high salinity and low soil moisture, which can affect stomatal activity [92]. Experiments have shown that C_3_ plants (*Salicornia europaea* ssp. and *Puccinellia nuttalliana*) native to highly saline marsh environments of Western Canada exhibit high δ^13^C values around −21.8‰ (adjusted for *Suess* effect), and vary between 6.5‰ and 4‰ across a 30m transect [92]. Halophyte plant communities can be highly nutritious sources of forage for domesticated livestock by providing a source of salt and other minerals that may be absent in traditional pastures [91]. We propose the strong synchronicity between δ^13^C and δ^18^O values may be a consequence of sheep/goat grazing on halophyte and/or vernal plant communities within the alluvial plain under a continental climatic regime.

The δ^15^N values of plants are also influenced by saline environments due to enrichment in soil ^15^N due to higher rates of denitrification that lead to ^15^N enriched soil nitrates [93]. Moderately high δ^15^N values (8.1±1‰) were found in caprine collagen in comparison to cattle (7.1±0.9‰). Enriched δ^15^N may be an indication of sheep/goat individuals grazing within saline soils, as has been seen in other studies [22]. On the other hand, it is possible that the ^15^N enrichment of bone collagen reflects animals visiting long-term pastures or grazing on crop by products under a manuring regime. Future studies are necessary to test this by determining the δ^15^N and δ^13^C values of remains of cereals and crop by-products.

During winter, the caprines from both sites exhibited low dietary δ^13^C values between −28.6‰ to −27.1‰, except for individual 7245 from Vinča-Belo Brdo. These values are comparable to ruminants dwelling in deciduous forested environments (−29.5‰ to −25.1‰) [94] and deer sampled from Vinča-Belo brdo (−29.7‰ to −25‰). Low plant foliar δ^13^C values can occur in forest with dense leaf cover caused by the canopy effect, which is caused by 13C depletion of atmospheric CO_2_ under the canopy due to uptake of recycled CO_2_ respired by ^13^C depleted organic matter [95], a decrease in light intensity near the forest floor decreasing efficiency of photosynthesis, discriminating against the transfer of ^13^C [83,84]. The net result is lower floral δ^13^C values in by 1 to 6‰ relative to floral growth in open environments, depending on canopy density which is dependent on tree species present in forests, the timing of the bud break, and leaf expansion [93,95,96]. In deciduous woodlands, canopy density is greatest during late spring/early summer, while non-deciduous forest stands retain its canopy throughout the year.

Charcoal analyses of Vinča-Belo brdo material indicate two main types of deciduous woodland environments were present near the settlement: riverine and hill slopes, with no large stands of non-deciduous trees [21]. The loss of leaves during autumn reduces the canopy, thus caprines grazing in woodland during winter will not result in low carbon values. However, provisioning animals with winter leaf hay collected during the summer months when the trees are in full leaf would result low δ^13^C values registering in caprine tissues. Another possibility is that leafy hay was collected from arboreal species native to riverine environments, such as ash (*Fraxinus excelsior*). Waterlogging caused by seasonal flooding has a similar impact on leaf carbon isotopes values as the canopy effect, (86]. Provisioning animals with leafy hay during winter may explain the large intra-tooth amplitude in δ^13^C values observed in caprines. Compound specific stable isotope analysis (CSIA) of amino acids (AA) extracted from dentine can test for the consumption woody plants. This is due to the difference in δ^15^N values of glutamine (Glx) and phenylamine (Phe) AA, which has been found to be significantly different between herbaceous and woody plants [97]. This method is independent of canopy effect principle concerning carbon isotope. When integrated with bioapatite analysis, it can verify winter leafy hay provisioning in bovids [98,99].

### Variation in cattle pasture and foddering practices

Overall, the results from the sequential analysis indicate strong inter-individual variation in cattle, unlike that previously observed in sheep teeth. Previous analysis proposed that domestic cattle from Vinča-Belo brdo grazed across different types of pastures while animals at Stubline were kept mainly on wooded or waterlogged pastures [22]. At Vinča-Belo brdo, dietary δ13C values from −28.2‰ to −22.1‰, based on bioapatite δ^13^C values exhibit a wider ranger greater than values previously observed for collagen (−26.4‰ to −23.0‰). This may reflect the attenuation of bone collagen stable isotope values due to bone turnover. The results from the sequential analysis indicate strong inter-individual variation in cattle from Vinča-Belo brdo, unlike that previously observed in sheep teeth.

Bos 7241 and Bos 7268 exhibit low δ^13^C values −26.1 to −24.5‰ with limited seasonal variation. However, three cattle individuals from Vinča-Belo brdo (Bos 7230, 7242 and 7244) exhibit δ^13^C values (>−24.1‰) as observed in caprine teeth. For Bos 7242 and 7244, these values occur in summer, as observed in caprine teeth. We propose this is the result of cattle grazing pastures supporting halophyte/vernal plant communities close to the sites, similar as proposed for caprines. For Bos 7230, high δ^13^C values reaching −21.1‰ during the period of winter season tooth formation may be evidence provisioning with hay collected from these vernal pastures in summer. There appears to be evidence for winter leaf hay provisioning in a single cattle, Bos 7240. This animal exhibits δ^13^C values as low as −28.2‰ during winter. Previous analyses of cattle from the LBK site of Bischoffsheim (Alsace [99]) and the 5^th^ millennium BC site of Bercy (France [25]) found cattle exhibited winter season δ^13^C values lower than *−*27.0‰. Considering that cattle from Vinča-Belo brdo and Stubline would have experienced weather conditions of a strong continental character (hot summers, cold winters), this lack of evidence for supplementary fodder is surprising given that during winter the snow cover as well as spring floods may have restricted access to fresh pastures.

Our study benefits from paleoenvironment evidence from the teeth of deer, which are typically forest dwelling. Dietary δ^13^C values for red deer between −29.7‰ and −25.0‰ are similar to the range of values for modern forest dwelling ruminants [94] and mid-Holocene aurochs from Boreal environments [100]. Carbon isotope values from individual 7240 fall within that of the deer dietary range. Leafy hay for cattle was collected from the same environment where the deer grazed would exhibit the same range of carbon isotopic values. It is also possible that Bos 7240 was also pastured within a woodland environment for the remaining part of the year. The dietary values from domesticated pigs (−27.2‰ to −25.6‰) were also within the deer range, thus potentially supporting the idea swine were managed under an open woodland pannage system for part of the year. The use of forest within the vicinity of Vinča-Belo brdo for pasture and pannage would support previous hypothesis proposed by Filipović, Marić [21] highlighting the diverse land use strategies practices by the Vinča communities.

### Vinča communities’ land-use

**Fig 6:**
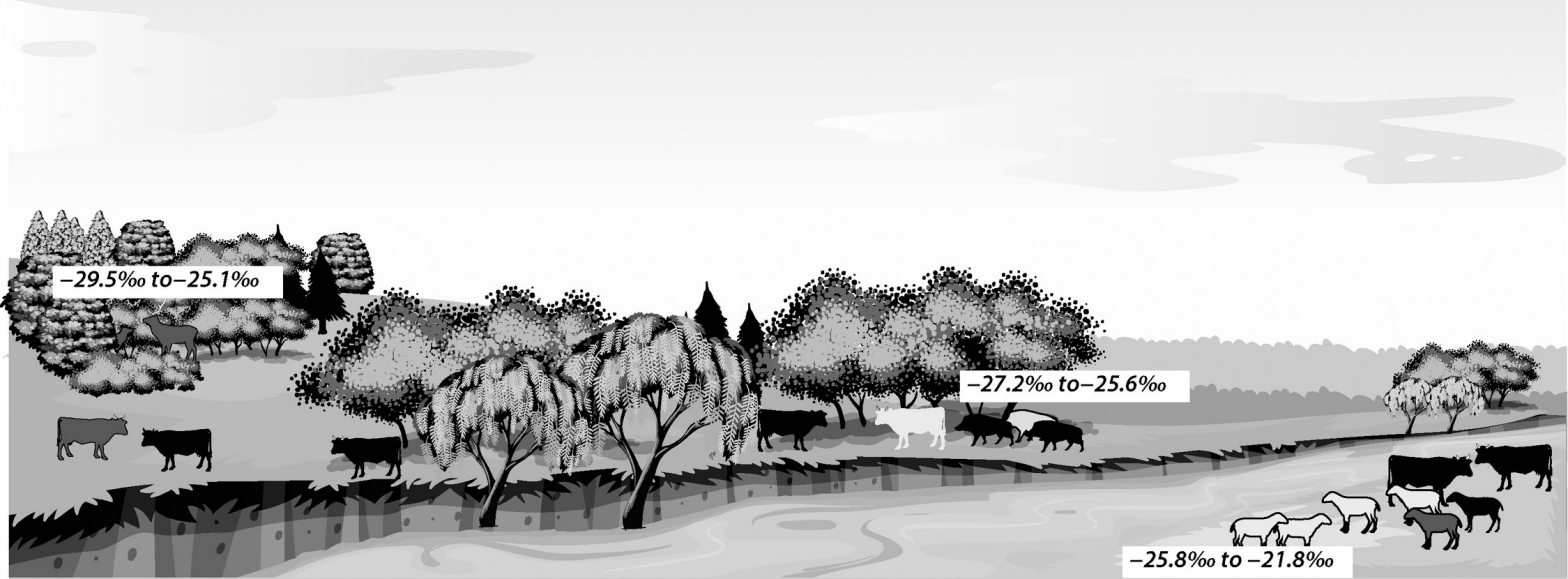
Illustration of the isotopic variation between ecosystems with the vicinity of Vinča-Belo brdo. The δ^13^C values indicate (from left to right): Deer and domesticated pigs sampled as part of this study, and Suess effected adjusted values for halophyte plants [92] to illustrate the gradient from dense to light forest canopies and salt marshes. The base images were made available on the ‘Nature scenes with fields’ https://www.vecteezy.com/vector-art/605564-nature-scenes-with-fields-and-mountians licensed to RG. The animal silhouettes are available under CC 4.0 from ArcheoZoo.org.

The results from incremental bioapatite samples from domesticated and wild animals of Vinča-Belo brdo and Stubline allow for nuanced investigation of the land use by late Vinča communities. Previous paleoenvironmental and zooarchaeological studies have proposed that these communities exploited a wide range of niches for wild resources and to raise their herds, particularly cattle [9,11,21,22]. The analysis presented here and elsewhere [22] demonstrates the wide range in environments exploited by communities living at Stubline and Vinča-Belo brdo (Fig 6). The seasonally available floodplain meadows appear to have played an important role in caprine management systems, and suggests sheep were not kept within settlement all year round as previously suggested [11,21]. This hypothesis was based on the principal that sheep grazing on riverine landscapes leaves animals susceptible to foot rot [11,21] caused by bacteria (Fusobacterium necrophorum/Dichelobacter nodosus). It leads to lameness severely limiting animal access to food [101]. Warm damp conditions found within permanent shelters promotes this condition, as do wet summers and warm winters, leading to a year-round endemic problem. Consequently, animals need to be grazed in large pastures and during winter, herders may have restricted grazing by penning animals within the settlement and provisioning animals leafy hay collected during late spring/summer to prevent foot rot and loss of sheep during floods.

The use of crop by-products and stubble for animal pasture and fodder may have also occurred. Archaeobotanical analysis of carbonised cereals and weed assemblages from Opovo, a contemporaneous site north of Vinča-Belo brdo, suggests that the majority of crops were sowed in the winter and harvested in the summer [102]. Allowing animals access after harvest would allow for manure to be directly distributed on cultivation plots. Crop plants may experience water stress in the final months prior to harvest and exhibit high δ^13^C values due to water stress [103], which may explain the δ^13^C values observed in sheep/goat individuals. The relatively high δ^15^N values found in sheep/goat bone collagen could reflect a foddering strategy where cereals were enriched with ^15^N due to manuring regimes [104,105].

Stable isotopic analysis of cattle teeth indicate several different husbandry strategies were applied. Some cattle exhibit δ^13^C and δ^18^O values similar to suggesting multi-species herding for all or part of the year (Fig 6), while other cattle exhibiting values more similar with cattle from contemporaneous LBK sites in Central Europe suggest both open grasslands or woodland grazing [106,107]. The large inter-individual variation seen in cattle may be due to different herding strategies applied to animals according to age and sex, use of more extensive pasturing strategies that evolved to cope with large herds, or grazing of small herds by individual households. Previous analysis of bone collagen values of sub-adult cattle (12 to 36 months) from at Popină Borduşani identified high variation in δ^13^C values between individuals. If these animals were raised for their meat, they may have been fed special diets to increase their weight or allowed to extensively range to enhance access to better quality pasture to increase their weight. The stable isotope values of cattle reflect diet during the formation of M3 (12–24 months). It is possible some of the individuals analysed here were male and this variation in δ^13^C values indicates animals being fattened for meat production.

Another source of carbon isotopic variation in cattle may be the emergence of extensive herding strategies where animals were able to roam between different habitats within the vicinity of the sites. This may represent a flexible and sustainable herding strategy where cattle were move naturally between pasture, reducing overgrazing. This type of management may only require one or two herdsmen that corrals individual household herds daily and moves them to pastures within the vicinity of the settlement. Collective herding strategies supports social cohesion and cooperation between households. Similar strategies are evident in traditional African societies, where village herds are graze together and returned to their owners in the evening for milking [108]. The similarity in carbon isotopes evident within caprines suggests a collective management of small stock herds from different households while the diversity in cattle stable isotopic results may also indicate different households/groups managing their animals individually.

Continual herd growth likely took place alongside increased intensity of settlement and population size in during the Late Vinca phases and would have placed significant pressure on local pasture resources. Due to the proximity of Vinča with other settlements and coupled with the increase in specialisation of animal products [11], herders may have negotiated access to pastures in exchange for consumables (milk/butter/cheese/meat) and trade goods as well as beasts. These individual efforts on herding of cattle may be motivated by increasing symbolic status of cattle associated with their considerable productive capacity to generate large quantities of meat, milk and skins, as well their ability to transform landscapes via traction [109]. This exchange may have resulted in the variation in stable isotope values found within the Vinča cattle. The negotiation and exchange of goods would of strengthened social relationships between Vinča communities [11] and the symbolic and economic importance for cattle. At the site of Opovo, Russell [110] proposes that over time, households may have built up their cattle herds, and this, coupled with the increasing representation of cattle in material culture, suggests the emergence of wealthy households [33]. Furthermore, she proposes that it may not have been possible to function in the Vinca social sphere without owning cattle due to its importance as bride wealth, blood money, sacrifices, or for feast [Russell 199:159]. Only through the integrated stable isotopic and zooarchaeological analysis of other Vinča settlements across time and space can we begin to explore cattle-based wealth systems and its potential impact on Vinča social relationships.

## Conclusions

Livestock herds were an important social and economic component of Vinča world and here we have uncovered the complex herding strategies employed for animal pasture and fodder. The combination of stable δ^13^C isotope values from different animal tissues has provided a means to reveal multi-faceted perspectives on herding strategies at different temporal scales (seasonal versus several years) thus highlighting the importance of multi-tissue analysis. Our study has uncovered the use by sheep flocks of seasonally available halophyte pastures and leafy hay, providing a new perspective on the sheep/goat management by Vinča communities. Future studies should consider including isotope analyses of botanical material to generate an integrated and detailed picture of herding and agricultural practices. There is also a need for detailed organic residues analysis of ceramic sherds to provide more information about processing of animal products.

The low variation in herding strategies for sheep/goat indicates a pooling of resources carried out a community level, while in contrast, the diversity in cattle pasturing strategies may be an indication of the emergence of household economies associated with the growing social and economic importance of cattle. This contrast in herding strategies between the two species highlights a level of complexity within the organisation of food procurement strategies for individual households and for the community in general. This study is limited to two sites, however, there is enormous potential for future stable isotopic studies considering the excellent preservation of faunal material from Vinča culture sites. More detailed analysis at other sites would enable a deeper understanding of Vinča culture herding practices as well as the landscape inhabited by these communities and their herds. Integrated with existing archaeological studies would provide a greater insight into the cultural evolution and the development of complex societies in Europe.

## Supporting information

S1 Table Sample list detailing context numbers, elements samples and age-at-death. (XLSX)

S2 Table Detailed carbon isotope results for individual teeth. (XLSX)

S3 Table Detailed oxygen isotope results for individual teeth. (XLSX)
