# Correction: Of herds and societies—Seasonal aspects of Vinča culture herding and land use practices revealed using sequential stable isotope analysis of animal teeth

**DOI:** 10.1371/journal.pone.0307585

**Published:** 2024-07-17

**Authors:** Rosalind E. Gillis, Jelena Bulatović, Kristina Penezić, Miloš Spasić, Nenad N. Tasić, Cheryl A. Makarewicz

The following information is missing from the Funding statement: The OA was supported by the Interdisciplinary Center for Archaeology and Evolution of Human Behaviour (ICArEHB), funded by the Portuguese Foundation for Science and Technology (FCT) under the program UIDP/04211/2020.

In Fig 3, “δ13C (filled diamond) and δ18O (open diamond) values for individual cattle teeth”, the panel I is a duplicate of panel H. Please view the correct Fig 3 here.

**Fig 3:**
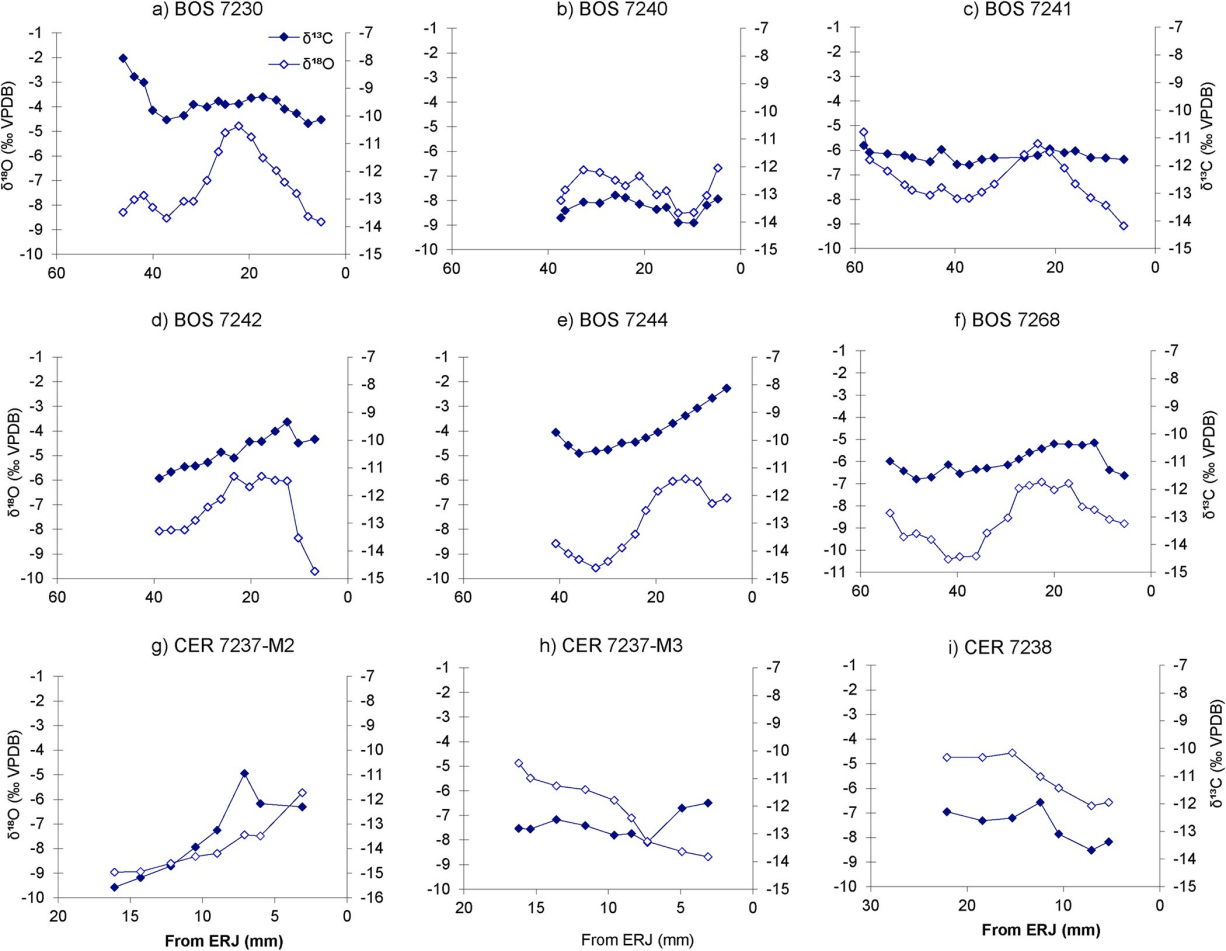
δ13C (filled diamond) and δ18O (open diamond) values for individual cattle teeth. Insets: a) Bos 7230, b) Bos 7240, c) Bos 7241, d) Bos 7242, e) Bos 7244 (Vinča-Belo brdo), f) Bos 7268 (Stubline), g) Cervus 7237-M2, h) Cervus 7237-M3 and i) Cervus 7238-M3. ERJ is the enamel root junction.

## References

[1] Gillis RE, Bulatović J, Penezić K, Spasić M, Tasić NN, Makarewicz CA (2021) Of herds and societies—Seasonal aspects of Vinča culture herding and land use practices revealed using sequential stable isotope analysis of animal teeth. PLOS ONE 16(10): e0258230. 10.1371/journal.pone.0258230 34618838 PMC8496836

